# Hepatitis C screening, diagnosis, and cascade of care among people aged > 40 years in Brasilia, Brazil

**DOI:** 10.1186/s12879-020-4809-2

**Published:** 2020-02-10

**Authors:** Daniela Mariano Carvalho-Louro, Eric Bassetti Soares, Jose Eduardo Trevizoli, Thayna Moreira Gomes Marra, Alexandre Lima Rodrigues da Cunha, Marcelo Palmeira Rodrigues, Adriana Claudia Lopes Carvalho-Furtado, Beatriz Taynara Araujo dos Santos, Francisco de Assis da Rocha Neves

**Affiliations:** 1 Gastroenterology Unit, Instituto Hospital de Base, Brasilia, Federal District 70322-000 Brazil; 2 Gilead Sciences Farmacêutica do Brasil Ltd. and Liver Center at UFMG, Federal University of Minas Gerais, Belo Horizonte, Minas Gerais 04711-130 Brazil; 3 Postgraduate Program in Health Sciences and Technologies, University of Brasilia, Brasilia, Federal District 70919-970 Brazil; 4 Sabin Laboratory Ltd., Brasilia, Federal District 70632-300 Brazil; 5 Pneumology Unit, Faculty of Medicine, University of Brasilia, Brasilia, Federal District 70673-432 Brazil; 6 Endocrinology Unit, Instituto Hospital de Base, Brasilia, Federal District 70322-000 Brazil; 7 Subsecretaria de Atencao Integral a Saude, Secretaria do Estado de Saude do Distrito Federal, Brasilia, Federal District 70770-200 Brazil; 8 Molecular Pharmacology Laboratory, Faculty of Health Sciences, University of Brasilia, Brasilia, Federal District 70919-970 Brazil

**Keywords:** Hepatitis C virus, Screening, Prevalence, Diagnosis, Chronic hepatitis C

## Abstract

**Background:**

Identifying patients with hepatitis C virus (HCV) infection and enhancing the cascade of care are essential for eliminating HCV infection. This study aimed to estimate the prevalence of positive anti-HCV serology in Brasilia, Brazil, and evaluate the efficiency of the cascade of care for HCV-positive individuals.

**Methods:**

This cross-sectional study analyzed 57,697 rapid screening tests for hepatitis C in individuals aged > 40 years between June 2018 and June 2019. HCV-positive patients were contacted and scheduled to undergo the HCV RNA viral test, genotyping, and transient elastography.

**Results:**

The prevalence of positive serology was 0.27%. Among 161 patients with positive anti-HCV serology, 124 (77%) were contacted, 109 (67.7%) were tested for HCV RNA viral load, and 69 (42.8%) had positive results. Genotype 1 (75%) was the most prevalent genotype. Among 65 patients (94.2%) who underwent transient elastography, 30 (46.2%) presented with advanced fibrosis. Additionally, of the 161 patients, 55 (34.1%) were referred for treatment, but only 39 (24.2%) complied, with 36 (22.4%) showing sustained virological response. By the end of the study, 16 patients were still awaiting to receive medication.

**Conclusions:**

The prevalence of HCV-positive patients was low in Brasilia, and the gaps in the cascade of care for these patients were significantly below the targets of HCV infection elimination. This study opens new avenues for eliminating HCV infection and suggests that partnerships with clinical laboratories to conduct anti-HCV tests are a useful strategy to improve HCV diagnosis.

**Trial registration:**

Research Ethics Committee of the Faculty of Health Sciences of the University of Brasília - UNB (CAAE number 77818317.2.0000.0030) and by the Ethics Committee of the Health Science Teaching and Research Foundation - FEPECS/SES/DF (CAAE number 77818317.2.3001.5553).

## Background

Chronic hepatitis C is a global public health problem, with a risk of progression to cirrhosis and hepatocellular carcinoma (HCC) [1, 2]. Hepatitis C is also associated with several comorbidities, such as cardiovascular and metabolic disease [3]. In 2017, an estimated 71 million people (about 1% of the world population) had active hepatitis C virus (HCV) infection, according to the Global Hepatitis Report by the World Health Organization (WHO) [4, 5]. Despite recent advances in hepatitis C treatment, the number of people with advanced liver disease and the number of liver disease-related deaths are predicted to increase in the coming years, [6]. with about 40% of liver cancers expected to be caused by HCV by 2030 [7]. To address this issue, it is crucial that the number of diagnoses and the access to treatment in most countries must be increased at least fivefold [8]. Currently, the main challenge is improving the diagnosis of chronic hepatitis C and having patients commence treatment to prevent liver cirrhosis and HCC [7, 9, 10]. Therefore, expanding the knowledge of HCV infection is essential to establish new HCV prevention and elimination programs in Brazil [4, 11].

A strategic plan has been outlined recently to address HCV infection in Brazil. It comprises incorporating new, effective, affordable drugs guiding physicians in treating HCV patients, and promoting diagnostic campaigns in populations aged > 40 years, as recent studies have shown a higher prevalence of infection [8, 12, 13]. Studies assessing HCV prevalence in Brazil are scarce, and the HCV seroprevalence is estimated between 0.69 to 1.89% [6, 13, 14]. The first Brazilian nationwide hepatitis survey demonstrated an HCV seroprevalence of 1.38% in 19,503 individuals [13]. It is postulated that the HCV prevalence in Brazil increased with age and is higher in adults born between 1950 and 1980, with genotype 1 most prevalent of chronic infections [6, 13, 14]. These studies confirmed Brazil as a country with low endemicity for hepatitis C.

Hepatitis C is commonly diagnosed by serological tests. Anti-HCV antibodies can be detected by immunological methods; however, these methods are costly and require long procedure times [15, 16]. In 2011, the US Food and Drug Administration approved the use of rapid tests (RTs) to detect HCV infection. RTs are easy to perform, yield faster results, and are cheaper [15]. Because RT shares a sensitivity and specificity similar to other HCV serological methods, they do not need a laboratory structure and are a great approach to increasing HCV diagnosis [17]. Considering an HCV seroprevalence of 0.71% in Brazil (based on a mathematical modeling approach), it would be necessary to increase the number of RTs performed annually to improve diagnosis and treatment and establish an HCV elimination program in the country [18].

With the high efficacy of new direct antiviral agents (DAAs), in Brazil, the challenge exists in need to identify and treat infected patients [19]. However, the lack of an efficient cascade of care for chronic HCV infection in Brazil hinders the diagnosis of the disease and access to treatment, which are the most critical factors for eliminating HCV infection and reducing HCV-related mortality [20]. This descriptive, observational, cross-sectional study aimed to evaluate the efficiency of the cascade of care for HCV-positive patients among people aged > 40 years in Brasilia, Brazil. Since we have examined a large population distributed throughout the Brasilia, Federal District, Brazil, we also decided to estimate the prevalence of positive anti-HCV serology by RT in these subjects.

## Methods

### Study setting

This study was conducted in Brasilia, Federal District, Brazil, which has a total population of approximately 2.974 million and an estimated aged > 40 years population of approximately 1.070 million, according to the Brazilian Institute of Geography and Statistics (IBGE) and the country’s public health agencies.

### Study subjects

This cross-sectional study was conducted between June 2018 and June 2019 as collaboration between the Brazilian Association of Hepatitis Carriers (ABPH) and the Sabin Laboratory. All subjects signed an informed consent form before participating in the study, in which the design design was approved by the Research Ethics Committee of the Faculty of Health Sciences of the University of Brasilia and the Ethics Committee of the Health Science Teaching and Research Foundation. Anti-HCV rapid tests (RTs) were performed free of charge for all individuals aged > 40 years at the Sabin Laboratory as screening tests to diagnose hepatitis C. The screening tests of the study subjects were carried out along with other requested blood tests. The Sabin Laboratory has several units in Brasilia, with an average attendance of approximately 500,000 people annually over the last 40 years, and holds international certifications, such as ISO 9001, ISO 14001, and ISO 31000, which attest to quality management processes.

### Inclusion criteria

Patients included in the study were adults aged over 40 years. HCV tests were performed voluntarily and when patients were submitted to any other type of blood laboratory test ordered by different physicians that were collected at several units of Sabin Laboratory in the Federal District between June 2018 and June 2019.

### Qualitative tests

The rapid anti-HCV test is an immunochromatographic qualitative test based on the use of captures antigens (core, NS3, NS4, and NS5) for selective identification of anti-HCV antibodies. If antibodies are present in the blood sample, they bind to capture antigen conjugates that are flowing through the membrane of the test device and are captured by antigens that are immobilized in the same area. This reaction produces a colored line in the test area. Conjugates that do not bind to the test area continue to flow and bind to immobilized reagents in the control area, also producing a colored line, demonstrating that the test reagents are working correctly [17, 21].

Both the WHO and the Brazilian Ministry of Health have established sensitivity (> 98% versus > 97%) and specificity (99.4% versus 99.4%) criteria to assess the quality of RTs for hepatitis C [22]. The Alere® rapid anti-HCV tests (Standard Diagnostics, South Korea; distributed by Bioeasy Diagnostica Ltda., Belo Horizonte and São Paulo, Brazil) were used in this study, and their sensitivity and specificity (100 and 92.7%, respectively) were compatible with the minimum criteria required [22, 23].

### Blood sample collection

Blood samples for the rapid anti-HCV test were collected by routine venipuncture during other ordered blood tests at the several units of the Sabin Laboratory in the Federal District. The samples were sent to the Sabin Laboratory headquarters, where each sample was centrifuged, and the serum was used to the test. RTs were performed, and a qualified biochemical professional interpreted the results in compliance with quality and safety standards. The tests were conducted according to manufacturer instructions [21, 22].

### Clinical investigation

Patients who tested positive were contacted by telephone and referred to the liver disease outpatient clinic of the Base Hospital Institute of the Federal District (IHBDF), a high-complexity public hospital in Brasilia, for consultation and further clinical investigation with HCV RNA viral load tests and genotyping using molecular biology techniques with real-time polymerase chain reaction (PCR). These tests were performed at the Central Public Health Laboratory of the Federal District or the Sabin Laboratory, and the results were retrieved from the electronic medical records. During the consultation, in addition to anamnesis and physical examination, transient elastography (TE) was performed.

Patients were considered lost to follow-up after three failed attempts to contact them by telephone or if they missed an appointment in the liver disease outpatient clinic at the IHBDF. Telephone calls were attempted at the time of the RT result, 30 days after the RT, and 3 months after the RT.

Liver fibrosis grade in patients was assessed by TE using Fibroscan® 502 (Echosens, Paris, France) during the hepatology consultations. Results of the liver stiffness measurement (LSM) were expressed in kilopascals (kPa), and those of the controlled attenuation parameter (CAP) measurement employed to quantify hepatic steatosis were described in decibels per meter (dB/m). The median values of 10 successful results of both measurements were obtained. A failure was defined as no single successful measurement, and an unreliable measurement was defined as an interquartile range to liver stiffness measurement ratio of > 0.30 [24–26].

### Statistical analysis

Epidemiological information, test results, and treatment indicated for each patient were grouped in a spreadsheet using Microsoft Office Excel 2013 software. All data, including age, sex, address, and results of complementary examinations, were categorized. Data were normally distributed, and continuous variables were expressed as means ± standard deviation, while categorical variables were expressed as percentages. The t-test for independent samples was used to compare means, while the chi-square test was used to compare proportions. Results were considered statistically significant when *p* < 0.05.

To estimate the sample size, it has been taken into account the estimated aged > 40 years population in Federal District (1.070 million), the margin of error of 0.4% and the confidence level of 95%. This resulted the number of 56.837 individuals to be analyzed as a minimum goal to be achieved.

The data were analyzed in SPSS for Mac OS X© (v.20.0.0; SPSS, Inc., Chicago, IL, USA).

## Results

### Estimated prevalence at the Federal District

Overall, 62,449 RTs were conducted as screening tests for hepatitis C in individuals aged > 40 years. Of these tests, 58,730 were performed in patients residing in the Federal District; the remaining 3719 RTs were excluded because they were executed in patients from other states, and 1033 RTs were excluded because they were performed inappropriately in patients aged < 40 years. Therefore, a total of 57,697 RTs was included in the study.

### Anti-HCV seroprevalence in the Federal District

In total, 181 patients had positive and valid RT results for HCV; among them, 161 resided in Brasilia, Federal District. Of the 161 patients, 124 (77%) were successfully contacted and were included in the study. It was not possible to contact the remaining 37 patients due to the lack of a valid telephone number or lack of response after three call attempts.

Considering the number of RTs performed in patients residing in the Federal District (57,697) and the number of HCV-positive patients (161), the prevalence found in this study was 0.27% (161/57,697).

In 21,291 RTs performed in male patients, 80 were positive (0.37%). Meanwhile, in 36,406 RTs performed in female patients, 81 were positive (0.22%), which was significantly lower than that in males (*p* < 0.001).

As shown in Table 1, HCV seroprevalence was higher in patients aged 50–69 years and lower in patients aged 40–49 years.

**Table 1:** Seroprevalence of HCV by age

Age	Positive results	HCV RTs*	Prevalence
40–49 years	28	17.211	0.16%
50–59 years	62	17.055	0.36%
60–69 years	48	13.416	0.35%
> 70 years	23	10.016	0.22%
TOTAL	161	57.698	0.27%

*HCV hepatitis C virus, *RTs* rapid tests

### Cascade of care

**Fig. 1:**
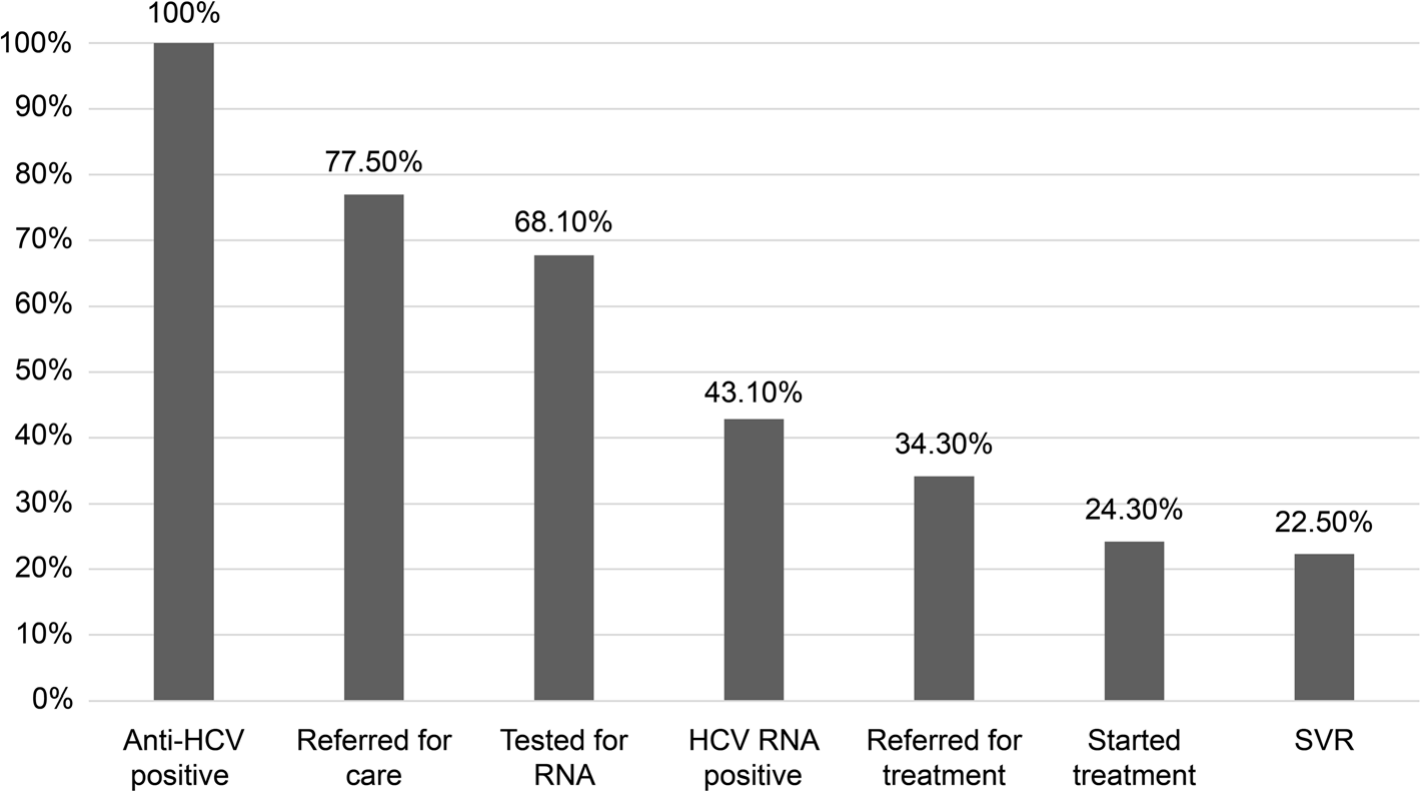
Cascade of care in patients aged > 40 years at a private laboratory in Federal District. HCV, hepatitis C virus; SVR, sustained virologic response

As shown in Fig. 1, of 161 patients with positive anti-HCV RTs, 124 (124/161 = 77%) were contacted by telephone and requested to attend a medical consultation at the IHBDF hepatology outpatient clinic. Among them, 109 (109/161 = 67.7%) attended the appointment. Moreover, all of them were tested for HCV RNA using PCR, and 69 (69/161 = 42.8%) were positive. As a means of continuing the cascade of care, all 69 patients were genotyped, and 65 were referred for TE using FibroScan®, only 4 patients did not attend Fibroscan®. A total of 55 (55/161 = 34.1%) patients were referred for clinical treatment with oral DAA regimens. Among them, 39 (39/161 = 24.2%) started treatment, but the remaining 16 did not because they were still waiting to receive medication at the end of the study. Of the patients who were on treatment, 36 (36/161 = 22.4%) had sustained virologic response (SVR) after 12 weeks of treatment. The SVR rate during the post-treatment follow-up at 12 weeks was 92.3% (36/39). One of the patients did not achieve SVR, other died during treatment due to cirrhosis complications, and another abandoned the study during the follow-up.

The other 20 patients with positive anti-HCV RT results who were not residents of the Federal District were contacted by telephone and referred to the specialized service for liver diseases at the IHBDF for clinical investigation and propaedeutic but were not included in the cascade of care.

**Fig. 2:**
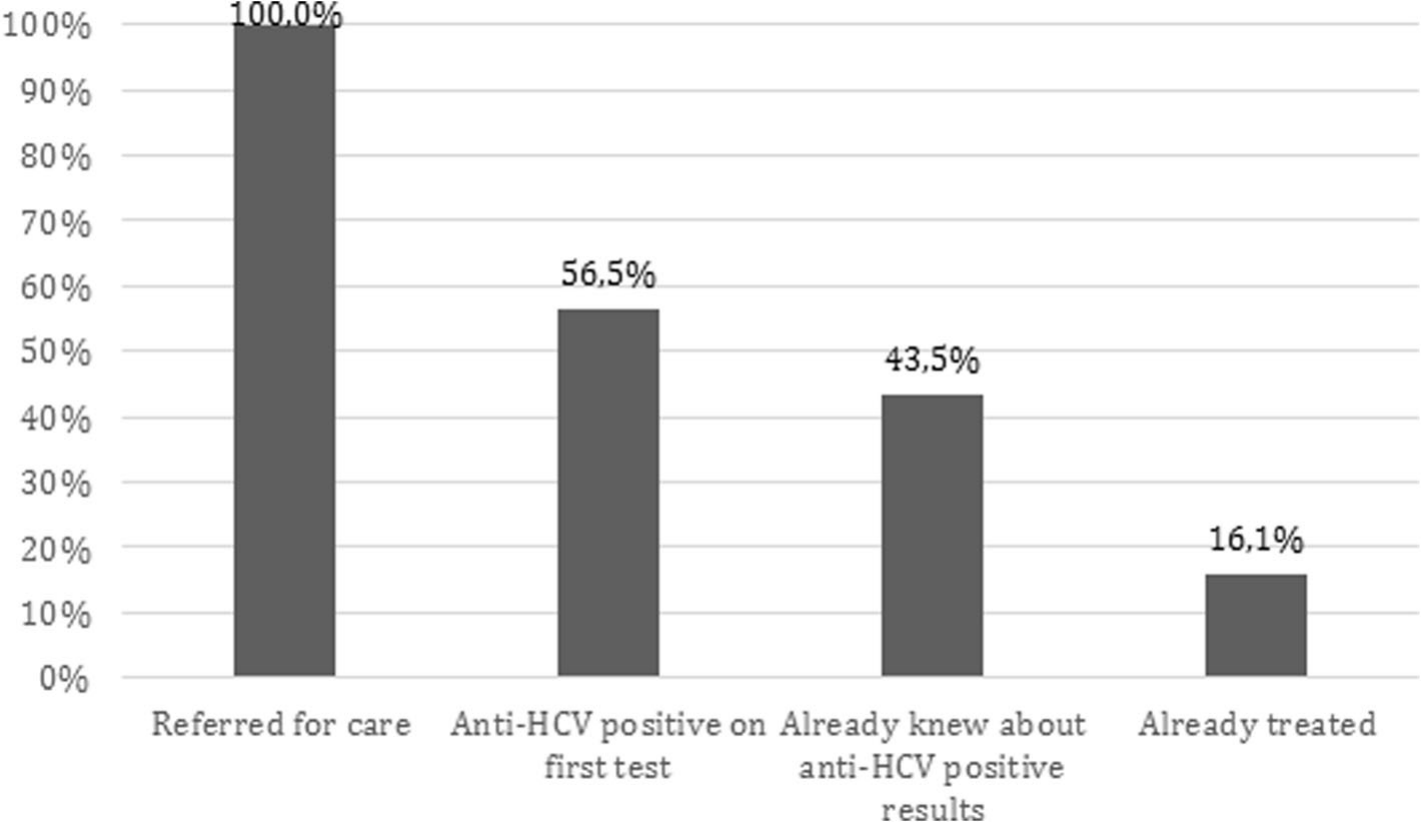
Patients percentage who started rapid anti-HCV test for first time versus previously positive serology results. HCV, hepatitis C vírus

Of the 124 patients with positive RTs who were contacted by telephone, 70 (70/124 = 56.5%) had never been screened for hepatitis C, and they expressed interest in continuing the investigation to evaluate possible chronic HCV infection, as shown in Fig. 2. On the other hand, 54 patients (54/124 = 43.5%) already knew of their positive anti-HCV serology results, and 20 (20/124 = 16.1%) were already being monitored and had already received treatment. These 54 HCV-positive patients were also invited to participate in the study to attend consultations for clinical investigation with HCV RNA viral load tests and genotyping. Patients, who presented with HCV-positive RNA, despite their knowledge of the disease and previous treatment, were referred for clinical treatment.

### Genotype results

Among the HCV genotypes from the 69 patients, genotype 1 (1a and 1b) was the most prevalent genotype (52 patients, 75%), followed by genotype 3 (11 patients, 16%) and genotype 2 (6 patients, 9%).

### Liver fibrosis grade and disease severity

A total of 65 patients (94.2%) with HCV-positive RNA tests were referred for TE to determine liver fibrosis grade and disease severity. Among them, 30 (30/65 = 46.2%) showed advanced fibrosis (F3 and F4), 12 (12/65 = 18.5%) showed significant fibrosis (F2), and 22 (22/65 = 33.8%) showed mild fibrosis (F0 and F1). In one patient (1.5%), the LSM by TE was not successful.

**Fig. 3:**
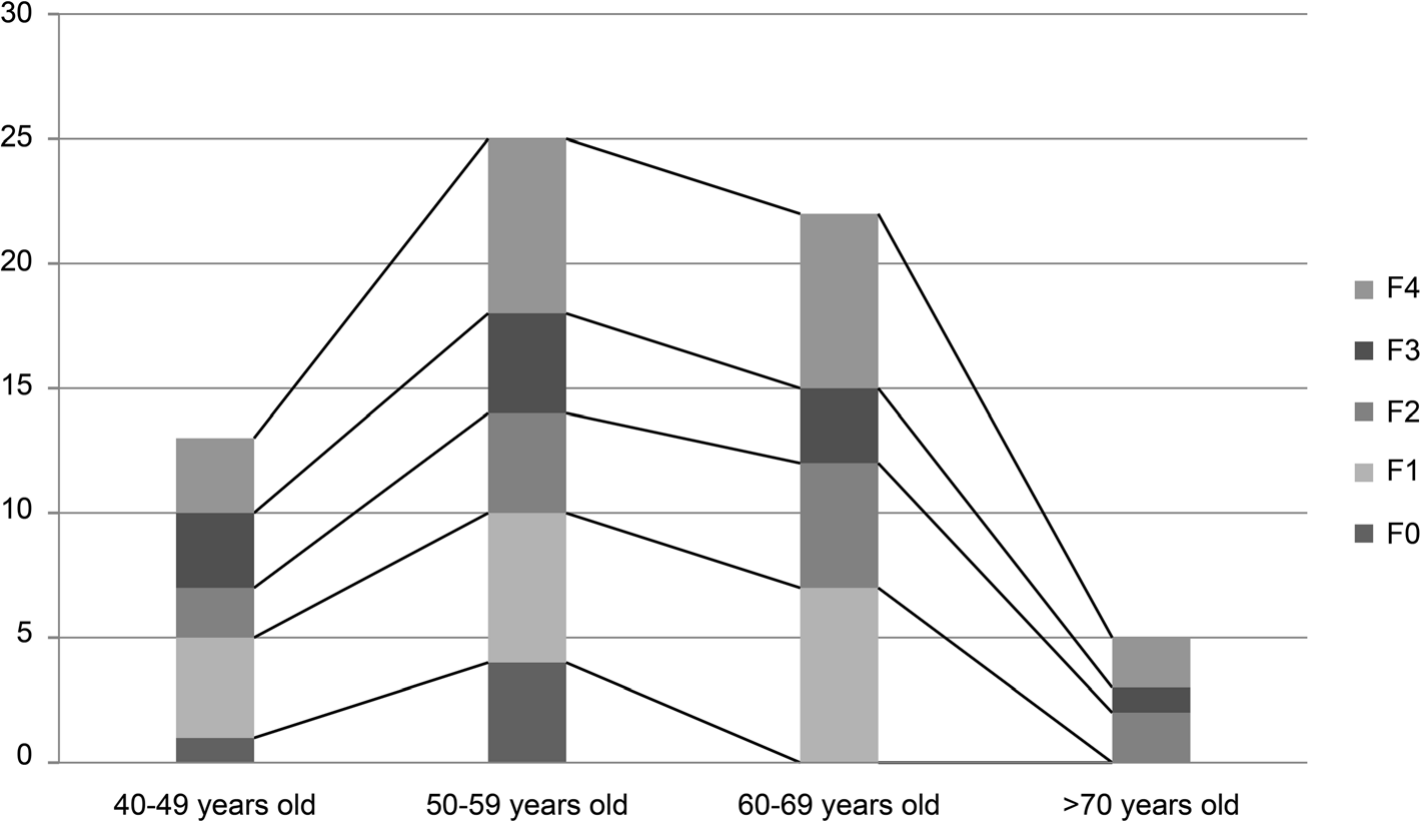
Degree of fibrosis according to age

As illustrated in Fig. 3, individuals aged > 70 years showed a higher prevalence of significant fibrosis (>F2) than those aged between 40 and 59 years.

To explore the relationship between genotype and severity of the liver disease, we analyzed the CAP and LSM values using FibroScan® according to genotype, as shown in Table 2. Because the genotype 2 categories consisted of only two patients, it was excluded from the analysis. Results of the t-test to compare genotype 1 with genotype 3 showed no significant difference. The distribution of patients with advanced fibrosis (F3 and F4) among all genotypes observed, also showed no statistically significant difference (*p* = 0.86), as shown in Table 3.

**Table 2:** LSM and CAP values according to genotype

	Genotype 1 (*n* = 48)	Genotype 3 (*n* = 10)	*p*
LSM*	11.8 ± 11.7	10.9 ± 5.4	0.45
CAP*	233 ± 45	221 ± 50	0.80

*CAP controlled attenuation parameter, *LSM* liver stiffness measurement

**Table 3:** Proportion of patients with advanced fibrosis according to genotype

	Advanced hepatic fibrosis (*n* = 43)	Nonadvanced hepatic fibrosis (*n* = 37)
Genotype 1	52.4%	47.6%
Genotype 2	50.0%	50.0%
Genotype 3	60.0%	40.0%

*p* = 0.86

## Discussion

This present study showed an estimated HCV prevalence of 0.27% among patients aged > 40 years, which is considered the risk group for HCV infection. This result displays a lower prevalence than was previously described by other studies in Brazil, which was between 0.69–1.89% [6, 13, 14]. In the Federal District, Pereira et al*l* demonstrated an HCV seroprevalence of 1.09% in 1008 individuals aged 20–69 years [13]. A mathematical modeling approach published in 2018 estimated that 700,000 people were chronically infected with HCV in Brazil and that the current prevalence of positive anti-HCV serology in the Brazilian population is 0.71%, as estimated by the Brazilian Ministry of Health [18]. The reduced prevalence of HCV infection in our study could have resulted from selecting individuals aged over 40 years. Notwithstanding, this interpretation is unlikely because previous studies conducted in Brazil have shown a higher prevalence in the adult population over 40 years [6, 13]. Pereira *et al* observed a prevalence of 0,75% in individuals between 10 and 19 years, 1,36% in 20–39 year, 1,55% in 40–59 years and 3,41% in those with 60–69 years [13]. Also, it is postulated that 76% of HCV chronic infection in Brazil occurs in adults born between 1950 and 1980 [6]. It is conceivable that the selection criteria can explain this difference. This study was not primarily designed to estimate the prevalence of HCV infection in the Federal District.

It is estimated that 71 million people are infected with HCV worldwide, and this infection is a leading cause of cirrhosis; hepatocellular carcinoma; and liver transplantation [27–29]. According to the Brazilian Ministry of Health, an estimated 700,000 individuals are chronically infected with HCV in Brazil [11, 18]. Most of these individuals would be aged > 40 years because previous studies demonstrated that to eliminate HCV according to WHO targets, the screening of HCV infection and the cascade of care should focus on patients born between 1958 and 1978 [30]. Furthermore, since HCV infection is curable, the progression rate of this disease is expected to expand significantly in the next 15 years, causing not only an increase in cirrhosis and liver cancer cases but also an increase in treatment costs [7]. To address this challenge, it is necessary to increase the rate of diagnosis and treatment in most countries [8].

Currently, a minority of HCV patients are diagnosed or are aware that they are infected with HCV and may inadvertently transmit the infection to other individuals [28, 29, 31]. Moreover, only 5–16% of chronic hepatitis C cases have been treated [32, 33]. According to the WHO, HCV infection elimination shall reduce new infections by 90% and diminish mortality by 65% until 2030 [28, 29].

This goal can be achieved by improving the diagnosis and efficiency of the cascade of care of hepatitis C patients [34]. Our study showed enormous gaps in the cascade of care of these patients, starting with the difficulty of patient access to medical care and finally with a low number of patients treated. Recent studies have also shown several gaps in the cascade of care for chronic HCV infection, including low diagnostic rate, difficulty in access to care for recently diagnosed patients, high cost of tests (such as the HCV RNA test), and low rate of treatment [32]. In the USA, a recent systematic review and meta-analysis to identify gaps in the care of HCV-positive patients showed that only 50% of all HCV-positive patients had been diagnosed. Additionally, only 43% of them had access to outpatient care, and 27% had HCV RNA confirmed. Moreover, only 16% received treatment, and 9% achieved SVR [32]. Similar results were also observed in European Union countries, where only 34% of infected individuals were diagnosed with chronic hepatitis C, and only 5% of all HCV cases were treated [23].

The exact reason for the lower HCV prevalence in this present study is unknown. However, the improvement in the cascade of care by the Brazilian healthcare system might have contributed to the diminishing HCV infection rate in the last 10 years. Another potential bias could be the experimental design of this study because blood samples were collected in a laboratory where most of the patients had private health insurance. Nevertheless, this study investigated a large-scale general population of 57,697 patients in the Federal District, including those regions with lower income per capita.

In Brazil, a cross-sectional retrospective study of HCV-infected blood donors showed that about 40% of them did not have access to a specialist and treatment, suggesting the need for improved access to medical care [35]. To the best of our knowledge, there has been no published research evaluating the cascade of care for patients aged > 40 years who have positive anti-HCV serology in Brazil, making this present study the first to demonstrate such an assessment of care. Our results were disappointed in the follow-up of HCV-positive patients since only 22.3% of them achieving SVR. Besides that, it is interesting to notice that the 16 patients were awaiting treatment at the end of the study. Since they were enrolled, three to 4 months after the end of the protocol, the medicines were dispensed to them. Despite these gaps, similar or even worse results were observed in other studies. A systematic review that examined the treatment cascade of patients with chronic hepatitis C in the USA identified several gaps that resulted in an SVR rate of only 5–6% [32]. In another study on the continuum of care of patients with HCV infection conducted in Philadelphia—which included HCV testing for homeless people and residents of public housing aged > 40 years—Coyle et al. performed HCV RNA tests using PCR in 89% of patients and 62.5% of them were examined by a hepatology specialist [33]. Furthermore, only 6.4% of their patients initiated anti-HCV treatment. However, they did not describe how many patients achieved SVR [33]. In our study, 92.3% of treated patients achieved SVR, and a high SVR rate was also recently noted in another study that evaluated the cascade of care for hepatitis C in this era of DAAs [36].

TE and other forms of fibrosis measurement replaced liver biopsy for the assessment of the degree of fibrosis, for a better selection of and patient referral for treatment and follow-up, as well as for early identification of HCC [24]. Of HCV-positive patients in this study almost half of them (30 patients, 46.2%) showed advanced fibrosis. A recent Brazilian study demonstrated that among 2000 HCV-infected patients in Brazil, 31.3% had cirrhosis, of which 29.1% of cases were F0/F1, 19.1% were F2, and 18.6% were F3. Most of them were evaluated by liver biopsy (61.5%), followed by liver elastography (24.5%) and clinical diagnosis of cirrhosis (13.9%) [37, 38]. Another Brazilian study reported that hepatic fibrosis increased with advancing age [37].

The most common genotypes in the present study were genotypes 1 (1a and 1b), 3, and 2, similar to those reported in several previous studies in Brazil, including a multicenter evaluation [13, 38–41].

This study has some limitations. First, the general representativeness of the population of the Federal District was limited because this study was not designed to estimate the prevalence of HCV infection in the Federal District and, the study was conducted in the 106 units of the biggest private laboratory in the Federal District. However, this is the most extensive screening test program that has been executed in all regions of the Federal District, including those with a lower income per capita. Second, as patients were referred to a single central hepatology service, among several others, in the Federal District, the loss to follow-up may also not reflect the overall existing losses in all regions of the Federal District. Third, selection criteria, recruiting only patients aged over 40 years, although being the most prevalent age group of infection, may result in a lower prevalence of infection in this study compared to other Brazilian studies. It is also important to remember that the Centers for Disease Control and Prevention (CDC) recommends that adult HCV screening should focus on individuals belonging to the birth cohort between 1945 and 1965 because studies have shown that two-thirds of HCV-infected individuals in the US belong to this age group. Fourth, there are other available Hep C screening tests, including the detection of hepatitis C virus RNA in saliva samples, although we have not yet available these tests for free population screening in Brazil.

According to the WHO, to eliminate global HCV infection by 2030, it is essential to develop new strategies to improve the rate of diagnosis and enhance treatment [28]. To achieve this target, 90% of people with chronic hepatitis C need to be diagnosed and 80% of them must be treated [29]. Moreover, such target can be achieved through the use of RTs for HCV infection screening [22, 28]. Meanwhile, the gaps identified in the cascade of care of patients in this study suggest the urgent need to develop an effective strategy for the screening, diagnosis, and treatment of HCV infection, which is aimed at eliminating the disease and reducing related mortality.

The lower prevalence observed in this study demonstrates that not only is the disease less common than initially thought, but it is also a challenge to identify patients with such viral infection. Our screening approach using anti-HCV RTs conducted at laboratories was feasible and can be implemented in collaboration with private and public laboratories. Because the blood sample collection was performed independent of RT requests and the RTs were carried out using a serum that was collected for other laboratory blood tests, the cost of this screening approach consisted only of the cost of the RT and the cost of performing the test. Trained health professionals can perform the test. This simple procedure can enhance the diagnosis of new cases of chronic hepatitis C, thereby supporting the global efforts of the WHO to eliminate hepatitis C by 2030. Furthermore, determining the HCV prevalence and the quality of the cascade of care provided new trends for the healthcare system to propose new strategies for patients with hepatitis C, including the acquisition of both RTs and drugs for HCV infection treatment.

## Conclusions

Our results revealed, in this sample, the lower prevalence of HCV than the others identified in previous studies and the similarity gaps in the cascade of care of patients with chronic HCV infection in Brazil to those of other developed countries, which is significantly below the targets of hepatitis C elimination. Our results also suggest that establishing partnerships with clinical laboratories to conduct rapid anti-HCV tests to detect and monitor disease is a cost-effective and feasible strategy that can be used to improve HCV diagnosis.

## Data Availability

The datasets used and/or analyzed during the current study are available from the corresponding author on reasonable request.

## Abbreviations

ABPH: Brazilian Association of Hepatitis Carriers
CAP: controlled attenuation parameter
DAA: direct antiviral agent
HCC: hepatocellular carcinoma
HCV: hepatitis C vírus
IBGE: Brazilian Institute of Geography and Statistics
IHBDF: Base Hospital Institute of the Federal District
LSM: liver stiffness measurement
PCR: polymerase chain reaction
RT: rapid test
SVR: sustained virologic response
TE: transient elastography
WHO: World Health Organization
